# Perivascular adipose tissue in vascular pathologies—a novel therapeutic target for atherosclerotic disease?

**DOI:** 10.3389/fcvm.2023.1151717

**Published:** 2023-05-26

**Authors:** Yusuke Adachi, Kazutaka Ueda, Eiki Takimoto

**Affiliations:** Department of Cardiovascular Medicine, Graduate School of Medicine, The University of Tokyo, Tokyo, Japan

**Keywords:** perivascular adipose tissue, beiging, brown adipose tissue, inflammation, atherosclerosis, remodeling

## Abstract

Most blood vessels are surrounded by adipose tissues known as perivascular adipose tissue (PVAT). Emerging experimental data have implicated the potential involvement of PVAT in the pathogenesis of cardiovascular disease: PVAT might be a source of inflammatory mediators under pathological conditions such as metabolic disorders, chronic inflammation, and aging, leading to vascular pathologies, while having vasculo-protective roles in a healthy state. PVAT has been also gaining attention in human disease conditions. Recent integrative omics approaches have greatly enhanced our understanding of the molecular mechanisms underlying the diverse functions of PVAT. This review summarizes recent progress in PVAT research and discusses the potential of PVAT as a target for the treatment of atherosclerosis.

## 1. Introduction

Classically, the pathogenesis of atherosclerosis has been considered to begin with damage to the intima of blood vessels (1–3), and endothelial cells have been regarded as the primary therapeutic target (4–6). Recent research, in turn, has demonstrated that perivascular adipose tissue (PVAT), the connective tissue surrounding blood vessels, substantially contributes to vascular physiological responses in an outside-in manner (7). Under non-pathological conditions, PVAT maintains vascular homeostasis and prevents progression of vascular inflammation and atherosclerosis. However, under pathological conditions, such as metabolic disorders, chronic inflammation, and aging, PVAT becomes a source of inflammatory mediators that exacerbate atherosclerosis (8). While the analysis of atherosclerotic plaque at the single-cell level has been performed (9, 10), integrative omics approaches involving PVAT could provide insights into a greater understanding of the pathophysiology of atherosclerosis and the molecular basis of the diverse functions of PVAT. Recently, glucagon-like peptide-1 receptor (GLP1R) agonists and sodium-glucose cotransporter-2 (SGLT2) inhibitors demonstrated cardiovascular benefits that might not be directly attributed to glycemic control (11). These agents have been shown to alter the quantity and quality of human coronary PVAT and epicardial adipose tissue, where PVAT might be potentially involved in such pathologies. In this review, we summarize recent advances in PVAT research and discuss the role of PVAT as a therapeutic target for atherosclerosis.

## 2. PVAT anatomy

Most arteries and veins with a diameter of 100 µm or greater are surrounded by PVAT, with a few exceptions, such as the cerebral arteries (12). PVAT is a connective tissue composed of adipocytes, preadipocytes, mesenchymal stem cells, fibroblasts, immune cells (macrophages, lymphocytes, and eosinophils), vascular cells, and nerves (13). PVAT has previously been referred to as epicardial adipose tissue (EAT), specifically pericoronary EAT for coronary arteries (11, 14), periaortic adipose tissue for the aorta (15), and visceral periadventitial adipose tissue for the mesenteric artery (16). Historically, PVAT was only considered a supportive tissue for blood vessels, but research conducted in 1991 on rat thoracic aorta revealed that vessels without PVAT had decreased reactivity to contractile stimuli such as epinephrine and electrical stimulation compared to vessels with PVAT, suggesting a potentially important role of PVAT in vascular reactivity (17).

## 3. PVAT characteristics

Recent studies have classified adipose tissues into white and brown adipose tissue based on function and morphology in mammals (18, 19). Functionally, white adipose tissue mainly stores energy in the form of triglycerides in cells and, when needed, supplies it to the blood as free fatty acids. Morphologically, white adipose tissue has only a small amount of cytoplasm and monocular lipid droplets, whereas brown adipose tissue has multilocular lipid droplets and a high concentration of mitochondria expressing uncoupling protein 1 (UCP1), a protein located in the inner mitochondrial membrane that produces heat instead of adenosine triphosphate (20, 21). The characteristics of PVAT vary based on animal species and anatomic region. In rodents, the mesenteric artery is surrounded by white adipose tissue, the thoracic aorta by brown adipose-like tissue, and the abdominal aorta by adipose tissue with a mixture of white and brown adipocytes (22). The coronary arteries in mice have no surrounding adipose tissue, whereas in humans and large laboratory animals, such as rabbits and pigs, these vessels are all surrounded by PVAT (22). As noted above, the human coronary PVAT is contained within EAT (11, 14).

## 4. PVAT as an endocrine organ

Recent studies have shown that adipose tissues function as endocrine organs (23). Adipocytes secrete a variety of cytokines, hormones, chemokines, and fatty acids referred to as adipokines (23). For example, PVAT secretes several pro-inflammatory factors such as interleukin-1β (IL-1β), interleukin-6 (IL-6), tumor necrosis factor-α (TNF-α), and monocyte chemotactic protein 1 (MCP-1), as well as anti-inflammatory factors such as adiponectin and nitric oxide (12, 14). Interestingly, the amount of adipokines secreted from PVAT does not correlate with the concentration of adipokines in plasma, suggesting the importance of the local autocrine or paracrine action of PVAT (14).

## 5. Function of PVAT in atherosclerosis

The removal of PVAT has been reported to significantly increase neointima formation after vascular injury, which was suppressed by transplanting healthy mouse subcutaneous adipose tissue, implying that PVAT has atheroprotective effects under non-pathological conditions (8). Consistently, selective deletion of peroxisome proliferator-activated receptor γ (PPARγ) specifically in vascular smooth muscle cells of mice has been reported to cause the loss of PVAT while simultaneously accelerating atherosclerosis (24), suggesting that the absence of healthy PVAT promotes the development of atherosclerosis. On the contrary, earlier studies have also shown that the pathological state of PVAT leads to atherosclerosis. High-fat and high-sucrose diet-induced obesity was found to cause inflammatory changes in the PVAT surrounding the femoral artery in mice, exacerbating the formation of atherosclerotic plaques after vascular injury (8). A study on the paired samples of EAT and subcutaneous adipose tissue collected during a coronary artery bypass graft surgery observed significantly higher levels of mRNA and protein expression of inflammatory cytokines (IL-1β, IL-6, MCP-1, and TNF-α) in EAT (14). These findings imply that PVAT in the pathological state promotes atherosclerosis and that PVAT is a source of inflammatory mediators in high-risk cardiac patients (14).

**Figure 1:**
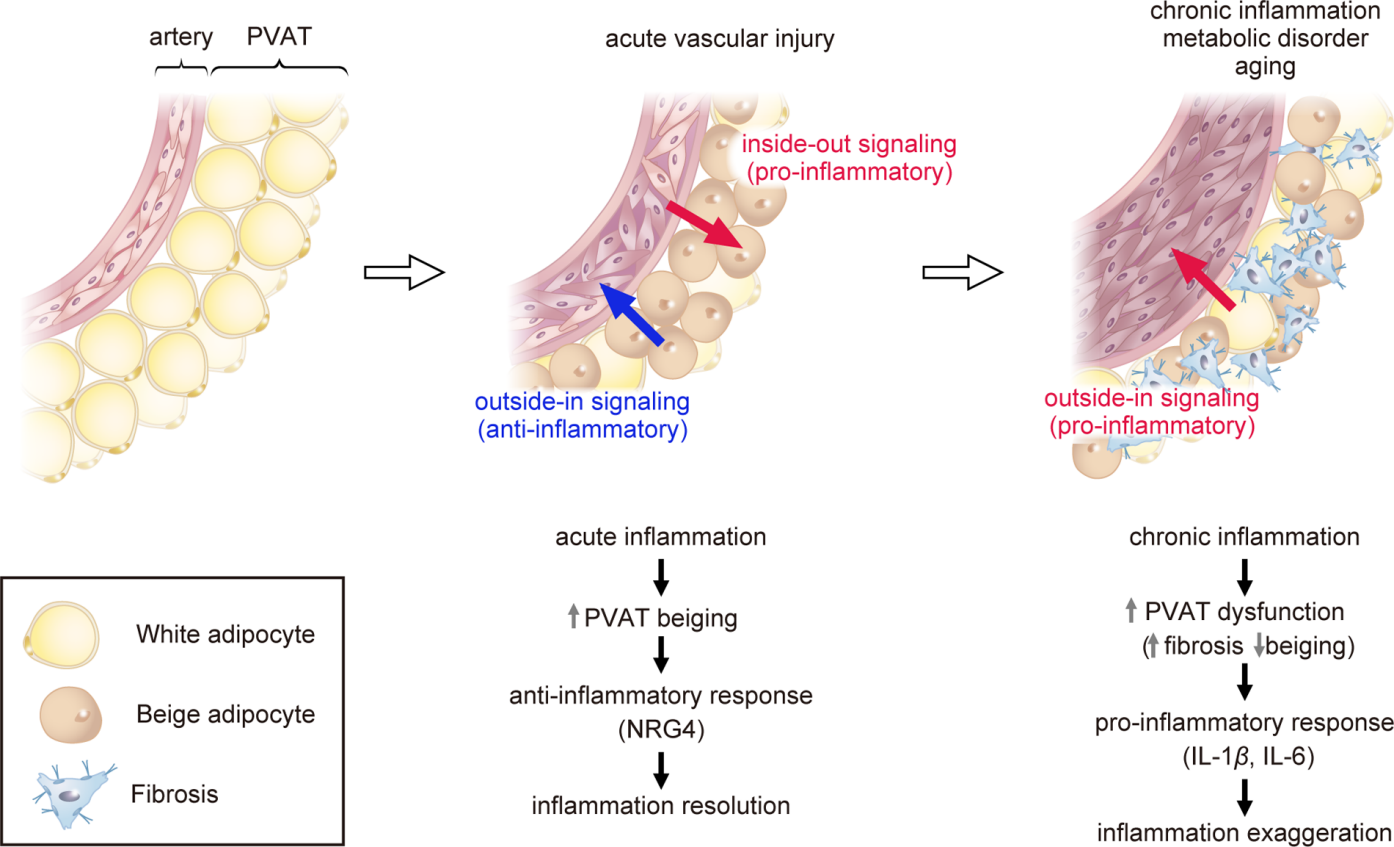
Role of perivascular adipose tissue (PVAT) in atherosclerosis. Acute vascular injury causes acute inflammation of PVAT (pro-inflammatory inside-out signaling) and triggers the process of “beiging” in PVAT as a protective mechanism. Beige PVAT then inhibits pathological vascular remodeling through the secretion of anti-inflammatory factors such as NRG4 (outside-in signaling). Chronic inflammation, metabolic disorder, or aging lead to PVAT dysfunction, promoting fibrosis adjacent to plaques and reducing the expression of beige adipose markers. Dysfunctional PVAT produces pro-inflammatory cytokines such as IL-1β and IL-6, exaggerating vascular inflammation (outside-in signaling). PVAT, perivascular adipose tissue; UCP1, uncoupling protein 1; NRG4, neuregulin 4; TGF-β, transforming growth factor-β; IL-1β, interleukin-1β; IL-6, interleukin-6.

Catecholamine or cold stimulation has been shown to convert white adipose tissue into beige adipose tissue, which expresses UCP1 and resembles brown adipose tissue morphologically. This phenomenon is referred to as “browning” or “beiging” (25, 26). Peroxisome proliferator-activated receptor gamma coactivator 1-alpha (PPARGC1A) and PR domain-containing 16 (PRDM16) are known as critical regulators of beiging (27–30). Studies in mouse models of atherosclerosis have shown that in the early stage of atherosclerosis, the beiging of PVAT is induced and serves to prevent the development of atherosclerotic plaques (31). In contrast, in the late stage of atherosclerosis, activation of the transforming growth factor-β (TGF-β) signaling pathway leads to fibrosis of the PVAT adjacent to plaques, resulting in PVAT dysfunction, decreased UCP1 expression, and increased PVAT inflammation (31). Studies using PVAT stromal cells (PVASCs) have shown that PPARGC1A is highly expressed in young mice PVASCs, but PPARGC1A expression in PVASCs decreases with age, resulting in decreased brown adipogenic differentiation (32). Recently, a decrease in the beiging of thoracic PVAT has been observed in an aging hypertensive rat model and has been linked to vascular dysfunction (33). The age-related loss of beiging capacity in PVAT may contribute to age-related vascular dysfunction (Figure 1) (32, 33).

We recently demonstrated that vascular injury induces PVAT beiging and suppresses vascular remodeling by modulating the inflammatory response (34). In a mouse model of vascular injury, macrophages accumulated in the PVAT and caused phenotypic changes of beiging. Silencing PRDM16, a key regulator to beiging, suppressed PVAT beiging and exacerbated inflammation and vascular remodeling following vascular injury. In contrast, the activation of PVAT beiging suppressed inflammation and pathological vascular remodeling via secretion of anti-inflammatory factors such as neuregulin 4 (NRG4), which critically regulate alternative macrophage activation. These findings suggest that PVAT beiging plays a pivotal anti-atherosclerotic role in the resolution of inflammation following vascular injury (Figure 1) (34).

## 6. Integrated omics analysis of PVAT

Recent studies using comprehensive analyses of PVAT uncovered the developmental process of PVAT and its diverse functions. Through single-cell transcriptomic analysis of perinatal PVAT from mice, Angueira et al. identified several distinct cell types, such as preadipocytes and mesenchymal progenitor cells (35). Subsequent cell differentiation assays and genetic lineage tracing studies revealed that the formation of PVAT in the aorta is mediated by fibroblast progenitor cells. In adult mice, the aortic adventitia was found to lack fibroblastic preadipocytes but contain adipogenic smooth muscle cells that contribute to adipocyte formation. These findings were mirrored in human adult PVAT samples analyzed using single-nucleus RNA sequencing, which revealed presumptive fibroblastic and smooth muscle-like adipocyte progenitor cells (35).

Pan et al. identified a high degree of transcriptional heterogeneity within PVASCs in the murine thoracic PVAT through single-cell RNA sequencing analysis of primary cultured PVASCs (Sca1^+^/CD90^+^/CD146^+^/CD29^+^) (32). They identified 10–12 distinct subpopulations with diverse lineages, including adipogenic, epithelial, neural, and endothelial lineages. Gene ontology analysis revealed that pathways related to vascular development were enriched in PVASCs, indicating a potential role in vascular remodeling. Additionally, human PVASCs were found to be involved in vascular remodeling and neointima formation upon transplantation into the mouse carotid artery ligation model. Gu et al. used single-cell RNA sequencing to demonstrate that mesenchymal stem cells expressing CD29^+^/Sca1^+^/PLIN1^−^/PECAM1^−^ (referred to as perivascular adipose tissue-derived stem cells, or PV-ADSCs) reside in PVAT and contribute to vascular remodeling through migration and smooth muscle cell differentiation (36). Using a vein graft model, the authors demonstrated that PV-ADSCs transplanted into the adventitia side of the vein graft significantly promote neointima formation through migration and differentiation to smooth muscle cells *in vivo*.

Our research group has recently discovered, through the utilization of bulk RNA sequencing, that PVAT undergoes beiging after acute vascular injury, which fine-tunes inflammatory response and thus vascular remodeling as a protective mechanism (34). Single-cell RNA sequencing further revealed that beige adipocytes abundantly express *Nrg4* (34). NRG4 is a member of the NRG protein family that acts via the ErbB receptor tyrosine kinase, and the NRG4-ErbB4 pathway is reported to suppress inflammation in macrophages (37, 38). Consistently, Shi et al. reported that NRG4 can inhibit endothelial inflammation through the ErbB4-protein kinase B (Akt)-nuclear factor (NF)-*κ*B pathway and protect against endothelial injury and atherosclerosis (39). These findings imply that the beiging of PVAT plays a crucial anti-atherosclerotic role in the resolution of inflammation following vascular injury, in part through the secretion of NRG4 (Figure 1) (34).

## 7. Effects of inflamed vessels on PVAT

Inflammation plays a significant role in the development and progression of atherosclerosis (2, 40, 41). Recent studies have shown that the inflammatory response induced by vascular damage expands into PVAT. In the mouse femoral artery wire injury model and rat iliac artery balloon injury model, vascular injury significantly upregulated the expression of inflammatory cytokines in PVAT (42). In a study using EAT specimens from patients undergoing cardiac surgery, macrophage infiltration and inflammatory cytokine expression were seen to be higher in the EAT of patients with coronary artery disease than in that of patients without coronary artery disease. The ratio of M1 (inflammatory) to M2 (anti-inflammatory) macrophages was positively correlated with the severity of coronary artery disease (43). Inflammatory cells accumulate in perivascular tissues prior to accumulation in the vessels after vascular injury, and inflammatory cell infiltration occurs from the outside to the inside of the vessel (34, 42). Vascular endothelial growth factor (VEGF), derived from adventitial fibroblasts, contributes to neointimal hyperplasia through vasa vasorum proliferation by activating the VEGFR2-extracellular signal-regulated kinase (ERK)1/2 signaling pathway (44), suggesting that adventitial fibroblasts play an important role in the infiltration of inflammatory cells from the outer to the inner vasculature.

18F-fluorodeoxyglucose positron emission tomography is a useful tool for assessing inflammation in adipose tissue, but it is expensive and not available in all healthcare facilities (45, 46). Recently, a non-invasive computed tomography angiography (CTA) technique was developed to quantify PVAT inflammation caused by vascular damage (47). In this method, the CT marker fat attenuation index (FAI) was determined by analyzing human coronary PVAT (EAT) obtained during coronary artery bypass graft surgery and was found to reflect adipose tissue inflammation. FAI measurements pertaining to human coronary arteries have a potential for detecting inflammation in acute coronary syndromes and vulnerable atherosclerotic plaques with reasonable sensitivity and specificity (47). In addition, a study involving 3,912 patients who underwent coronary CTA found that peri-coronary FAI was able to predict cardiac risk more effectively than traditional coronary risk factors (48). Furthermore, peri-coronary FAI levels were significantly higher in patients with coronary spastic angina compared to those without, implying that perivascular inflammation may play a role in the development of coronary spasm (49). In patients with abdominal aortic aneurysms, high periaortic FAI levels were also found to be an independent predictor of aortic aneurysm progression (50). However, perivascular inflammation can be present even in the absence of plaque, raising the question of what comes first (51). It is challenging to discern between the inside-out vs. outside-in issues. This noninvasive approach to appraising PVAT inflammation by CTA may aid in predicting atherosclerotic stability and the development of cardiovascular events, but further evaluation is required to determine its efficacy.

## 8. PVAT/EAT as a potential therapeutic target

We recently demonstrated that local activation of beiging in mouse femoral artery PVAT using a pluronic gel containing CL316243, a specific β3-adrenergic receptor agonist, suppressed inflammation and vascular remodeling after vascular injury (34). Another study confirmed that local injection of human PVASC, which overexpresses *Ppargc1a* to promote beiging, into the PVAT after ligation of mouse carotid arteries significantly reduced perivascular collagen deposition (32). These results suggest that the beiging activation of PVAT may be a promising therapeutic target for atherosclerosis. However, some research indicates that systemic activation of beiging exacerbates atherosclerosis via brown fat-mediated lipolysis (52), suggesting that the anti-atherosclerotic effects of beiging activation may differ depending on which adipose tissue is targeted for beiging.

Recent cardiovascular clinical trials have demonstrated that antidiabetic medications can provide cardiovascular benefits beyond glycemic control. GLP1R agonists, which are used to treat type 2 diabetes and obesity, have been shown to lower the incidence of cardiovascular death, myocardial infarction, and stroke (53–55). GLP1R agonists have been found to reduce EAT thickness more effectively than overall weight loss in patients with type 2 diabetes and obesity (56–58). Notably, GLP1R is expressed in the EAT but not in subcutaneous adipose tissue (59), suggesting that GLP1R agonists may directly impact PVAT/EAT. While studies in rats have indicated that GLP1R agonists stimulate the thermogenic activity of brown adipose tissue via hypothalamic AMP-activated protein kinase (60), they may also exert their effects through other pathways. Dozio et al. collected EAT from patients with coronary artery disease undergoing coronary artery bypass graft surgeries and performed microarray analysis to show that GLP1R expression in the EAT was positively correlated with the expression of genes that induce white-to-brown adipose tissue differentiation (61). GLP1R agonists have been proposed to target GLP1R in the EAT to inhibit local adipogenesis, enhance fat utilization, and induce brown adipose tissue differentiation (11, 61).

SGLT2 inhibitors, which are used to treat heart failure regardless of diabetes status, have been shown in cardiovascular outcome trials to reduce the risk of major adverse cardiovascular events, cardiovascular death, and heart failure (62, 63). SGLT2 inhibitors such as dapagliflozin and empagliflozin have significantly reduced the EAT thickness or volume in humans (64–69). In mice with diet-induced obesity, SGLT2 inhibition by empagliflozin promotes fat utilization and beiging of adipose tissue while decreasing inflammation and insulin resistance by polarizing macrophages in adipose tissue to an anti-inflammatory state (70–72). The SGLT2 inhibitor luseogliflozin suppresses neointimal hyperplasia after vascular injury in high-fat diet-fed mice, partly by suppressing macrophage platelet-derived growth factor subunit B expression in the PVAT (73). Further research is needed to determine the independent effects of SGLT2 inhibitors on human PVAT/EAT.

## 9. Limitations and future direction of PVAT research

Establishing causality in the relationship between inflamed vessels and adjacent fat inflammation and signaling is challenging and may be confounded by outside-in signaling. While current evidence suggests a bidirectional relationship between these phenomena, further research is necessary to determine the directionality and underlying mechanisms. Additionally, while existing data from mouse studies supports the hypothesis that PVAT is involved in atherosclerosis, normal mice differ from humans in susceptibility to atherosclerosis and vascular remodeling. Therefore, we need to be careful when applying results obtained in mice to humans. Further research is necessary to establish the clinical relevance of PVAT and to identify potential therapeutic targets for human vascular disease. Finally, while the current data support the hypothesis that GLP1R agonists and SGLT2 inhibitors may target PVAT/EAT to exert cardiovascular protection, it is important to note that this conclusion is based on limited evidence and may be subject to alternative interpretations. Previous investigations have suggested that these agents can induce generalized weight loss, improve insulin sensitivity, reduce blood pressure, lower lipid levels, and decrease visceral and epicardial adiposity (62, 63, 74–76). To fully elucidate the mechanisms underlying these effects and their potential relevance to PVAT/EAT, further research is warranted.

## 10. Conclusion

Recent studies have shown that PVAT plays a crucial role in maintaining vascular homeostasis and preventing the development of atherosclerosis. Integrated omics analyses have revealed that PVAT undergoes a process known as “beiging” after acute vascular injury, which serves as a protective mechanism by inhibiting pathological vascular remodeling through the secretion of anti-inflammatory factors such as NRG4. However, chronic inflammation, metabolic disorder, or aging can lead to PVAT dysfunction, resulting in fibrosis adjacent to plaques and reduced PVAT beiging in the advanced stages of atherosclerosis. Future studies targeting PVAT will open a new avenue for treating atherosclerosis.

## References

[1] Ross R Glomset JA. The pathogenesis of atherosclerosis. N Engl J Med. (1976) 295:369–77. 10.1056/nejm197608122950707 819830

[2] Rader DJ Daugherty A. Translating molecular discoveries into new therapies for atherosclerosis. Nature. (2008) 451:904–13. 10.1038/nature06796 18288179

[3] Silvestre-Roig C Braster Q Ortega-Gomez A Soehnlein O. Neutrophils as regulators of cardiovascular inflammation. Nat Rev Cardiol. (2020) 17:327–40. 10.1038/s41569-019-0326-7 31996800

[4] Gimbrone MA Jr García-Cardeña G. Endothelial cell dysfunction and the pathobiology of atherosclerosis. Circ Res. (2016) 118:620–36. 10.1161/circresaha.115.306301 26892962 PMC4762052

[5] Libby P Ridker PM Hansson GK. Inflammation in atherosclerosis: from pathophysiology to practice. J Am Coll Cardiol. (2009) 54:2129–38. 10.1016/j.jacc.2009.09.009 19942084 PMC2834169

[6] Aikawa M Sugiyama S Hill CC Voglic SJ Rabkin E Fukumoto Y Lipid lowering reduces oxidative stress and endothelial cell activation in rabbit atheroma. Circulation. (2002) 106:1390–6. 10.1161/01.cir.0000028465.52694.9b 12221058

[7] Kim HW Shi H Winkler MA Lee R Weintraub NL. Perivascular adipose tissue and vascular perturbation/atherosclerosis. Arterioscler Thromb Vasc Biol. (2020) 40:2569–76. 10.1161/ATVBAHA.120.312470 32878476 PMC7577939

[8] Takaoka M Nagata D Kihara S Shimomura I Kimura Y Tabata Y Periadventitial adipose tissue plays a critical role in vascular remodeling. Circ Res. (2009) 105:906–11. 10.1161/CIRCRESAHA.109.199653 19762682

[9] Depuydt MAC Prange KHM Slenders L Ord T Elbersen D Boltjes A Microanatomy of the human atherosclerotic plaque by single-cell transcriptomics. Circ Res. (2020) 127:1437–55. 10.1161/CIRCRESAHA.120.316770 32981416 PMC7641189

[10] Emoto T Yamamoto H Yamashita T Takaya T Sawada T Takeda S Single-cell RNA sequencing reveals a distinct immune landscape of myeloid cells in coronary culprit plaques causing acute coronary syndrome. Circulation. (2022) 145:1434–6. 10.1161/CIRCULATIONAHA.121.058414 35500048

[11] Iacobellis G. Epicardial adipose tissue in contemporary cardiology. Nat Rev Cardiol. (2022) 19:593–606. 10.1038/s41569-022-00679-9 35296869 PMC8926097

[12] Henrichot E Juge-Aubry CE Pernin A Pache JC Velebit V Dayer JM Production of chemokines by perivascular adipose tissue: a role in the pathogenesis of atherosclerosis? Arterioscler Thromb Vasc Biol. (2005) 25:2594–9. 10.1161/01.ATV.0000188508.40052.35 16195477

[13] Gil-Ortega M Somoza B Huang Y Gollasch M Fernandez-Alfonso MS. Regional differences in perivascular adipose tissue impacting vascular homeostasis. Trends Endocrinol Metab. (2015) 26:367–75. 10.1016/j.tem.2015.04.003 26008879

[14] Mazurek T Zhang L Zalewski A Mannion JD Diehl JT Arafat H Human epicardial adipose tissue is a source of inflammatory mediators. Circulation. (2003) 108:2460–6. 10.1161/01.CIR.0000099542.57313.C5 14581396

[15] Police SB Thatcher SE Charnigo R Daugherty A Cassis LA. Obesity promotes inflammation in periaortic adipose tissue and angiotensin II-induced abdominal aortic aneurysm formation. Arterioscler Thromb Vasc Biol. (2009) 29:1458–64. 10.1161/ATVBAHA.109.192658 19608970 PMC2753598

[16] Verlohren S Dubrovska G Tsang SY Essin K Luft FC Huang Y Visceral periadventitial adipose tissue regulates arterial tone of mesenteric arteries. Hypertension. (2004) 44:271–6. 10.1161/01.HYP.0000140058.28994.ec 15302842

[17] Soltis EE Cassis LA. Influence of perivascular adipose tissue on rat aortic smooth muscle responsiveness. Clin Exp Hypertens A. (1991) 13:277–96. 10.3109/10641969109042063 2065467

[18] Harms M Seale P. Brown and beige fat: development, function and therapeutic potential. Nat Med. (2013) 19:1252–63. 10.1038/nm.3361 24100998

[19] Virtanen KA Lidell ME Orava J Heglind M Westergren R Niemi T Functional brown adipose tissue in healthy adults. N Engl J Med. (2009) 360:1518–25. 10.1056/NEJMoa0808949 19357407

[20] Kajimura S Spiegelman BM Seale P. Brown and beige fat: physiological roles beyond heat generation. Cell Metab. (2015) 22:546–59. 10.1016/j.cmet.2015.09.007 26445512 PMC4613812

[21] Wang W Seale P. Control of brown and beige fat development. Nat Rev Mol Cell Biol. (2016) 17:691–702. 10.1038/nrm.2016.96 27552974 PMC5627770

[22] Brown NK Zhou Z Zhang J Zeng R Wu J Eitzman DT Perivascular adipose tissue in vascular function and disease: a review of current research and animal models. Arterioscler Thromb Vasc Biol. (2014) 34:1621–30. 10.1161/ATVBAHA.114.303029 24833795 PMC4104287

[23] Skurk T Alberti-Huber C Herder C Hauner H. Relationship between adipocyte size and adipokine expression and secretion. J Clin Endocrinol Metab. (2007) 92:1023–33. 10.1210/jc.2006-1055 17164304

[24] Chang L Villacorta L Li R Hamblin M Xu W Dou C Loss of perivascular adipose tissue on peroxisome proliferator-activated receptor-gamma deletion in smooth muscle cells impairs intravascular thermoregulation and enhances atherosclerosis. Circulation. (2012) 126:1067–78. 10.1161/CIRCULATIONAHA.112.104489 22855570 PMC3493564

[25] Wu J Bostrom P Sparks LM Ye L Choi JH Giang AH Beige adipocytes are a distinct type of thermogenic fat cell in mouse and human. Cell. (2012) 150:366–76. 10.1016/j.cell.2012.05.016 22796012 PMC3402601

[26] Sidossis L Kajimura S. Brown and beige fat in humans: thermogenic adipocytes that control energy and glucose homeostasis. J Clin Invest. (2015) 125:478–86. 10.1172/JCI78362 25642708 PMC4319444

[27] Dempersmier J Sambeat A Gulyaeva O Paul SM Hudak CS Raposo HF Cold-inducible Zfp516 activates UCP1 transcription to promote browning of white fat and development of brown fat. Mol Cell. (2015) 57:235–46. 10.1016/j.molcel.2014.12.005 25578880 PMC4304950

[28] Seale P Bjork B Yang W Kajimura S Chin S Kuang S PRDM16 controls a brown fat/skeletal muscle switch. Nature. (2008) 454:961–7. 10.1038/nature07182 18719582 PMC2583329

[29] Ohno H Shinoda K Spiegelman BM Kajimura S. PPARgamma agonists induce a white-to-brown fat conversion through stabilization of PRDM16 protein. Cell Metab. (2012) 15:395–404. 10.1016/j.cmet.2012.01.019 22405074 PMC3410936

[30] Cohen P Levy JD Zhang Y Frontini A Kolodin DP Svensson KJ Ablation of PRDM16 and beige adipose causes metabolic dysfunction and a subcutaneous to visceral fat switch. Cell. (2014) 156:304–16. 10.1016/j.cell.2013.12.021 24439384 PMC3922400

[31] Kim S Lee ES Lee SW Kim YH Lee CH Jo DG Site-specific impairment of perivascular adipose tissue on advanced atherosclerotic plaques using multimodal nonlinear optical imaging. Proc Natl Acad Sci U S A. (2019) 116:17765–74. 10.1073/pnas.1902007116 31427531 PMC6731664

[32] Pan XX Ruan CC Liu XY Kong LR Ma Y Wu QH Perivascular adipose tissue-derived stromal cells contribute to vascular remodeling during aging. Aging Cell. (2019) 18:e12969. 10.1111/acel.12969 31087498 PMC6612678

[33] Kong LR Zhou YP Chen DR Ruan CC Gao PJ. Decrease of perivascular adipose tissue browning is associated with vascular dysfunction in spontaneous hypertensive rats during aging. Front Physiol. (2018) 9:400. 10.3389/fphys.2018.00400 29720945 PMC5915562

[34] Adachi Y Ueda K Nomura S Ito K Katoh M Katagiri M Beiging of perivascular adipose tissue regulates its inflammation and vascular remodeling. Nat Commun. (2022) 13:5117. 10.1038/s41467-022-32658-6 36071032 PMC9452496

[35] Angueira AR Sakers AP Holman CD Cheng L Arbocco MN Shamsi F Defining the lineage of thermogenic perivascular adipose tissue. Nat Metab. (2021) 3(4):469–84. 10.1038/s42255-021-00380-0 33846639 PMC8136151

[36] Gu W Nowak WN Xie Y Le Bras A Hu Y Deng J Single-cell RNA-sequencing and metabolomics analyses reveal the contribution of perivascular adipose tissue stem cells to vascular remodeling. Arterioscler Thromb Vasc Biol. (2019) 39:2049–66. 10.1161/ATVBAHA.119.312732 31340667 PMC6766361

[37] Schumacher MA Hedl M Abraham C Bernard JK Lozano PR Hsieh JJ Erbb4 signaling stimulates pro-inflammatory macrophage apoptosis and limits colonic inflammation. Cell Death Dis. (2017) 8:e2622. 10.1038/cddis.2017.42 28230865 PMC5386486

[38] Schumacher MA Dennis IC Liu CY Robinson C Shang J Bernard JK NRG4-ErbB4 signaling represses proinflammatory macrophage activity. Am J Physiol Gastrointest Liver Physiol. (2021) 320:G990–G1001. 10.1152/ajpgi.00296.2020 33826403 PMC8285586

[39] Shi L Li Y Xu X Cheng Y Meng B Xu J Brown adipose tissue-derived Nrg4 alleviates endothelial inflammation and atherosclerosis in male mice. Nat Metab. (2022) 4:1573–90. 10.1038/s42255-022-00671-0 36400933 PMC9684073

[40] Ruparelia N Chai JT Fisher EA Choudhury RP. Inflammatory processes in cardiovascular disease: a route to targeted therapies. Nat Rev Cardiol. (2017) 14:133–44. 10.1038/nrcardio.2016.185 27905474 PMC5525550

[41] Libby P Ridker PM Hansson GK. Progress and challenges in translating the biology of atherosclerosis. Nature. (2011) 473:317–25. 10.1038/nature10146 21593864

[42] Takaoka M Suzuki H Shioda S Sekikawa K Saito Y Nagai R Endovascular injury induces rapid phenotypic changes in perivascular adipose tissue. Arterioscler Thromb Vasc Biol. (2010) 30:1576–82. 10.1161/ATVBAHA.110.207175 20489168

[43] Hirata Y Tabata M Kurobe H Motoki T Akaike M Nishio C Coronary atherosclerosis is associated with macrophage polarization in epicardial adipose tissue. J Am Coll Cardiol. (2011) 58:248–55. 10.1016/j.jacc.2011.01.048 21737014

[44] Li XD Hong MN Chen J Lu YY Ye MQ Ma Y Adventitial fibroblast-derived vascular endothelial growth factor promotes vasa vasorum-associated neointima formation and macrophage recruitment. Cardiovasc Res. (2020) 116:708–20. 10.1093/cvr/cvz159 31241138

[45] Christen T Sheikine Y Rocha VZ Hurwitz S Goldfine AB Di Carli M Increased glucose uptake in visceral versus subcutaneous adipose tissue revealed by PET imaging. JACC Cardiovasc Imaging. (2010) 3:843–51. 10.1016/j.jcmg.2010.06.004 20705265 PMC4042675

[46] Bucerius J Mani V Wong S Moncrieff C Izquierdo-Garcia D Machac J Arterial and fat tissue inflammation are highly correlated: a prospective 18F-FDG PET/CT study. Eur J Nucl Med Mol Imaging. (2014) 41:934–45. 10.1007/s00259-013-2653-y 24442596 PMC4024167

[47] Antonopoulos AS Sanna F Sabharwal N Thomas S Oikonomou EK Herdman L Detecting human coronary inflammation by imaging perivascular fat. Sci Transl Med. (2017) 9(398):eaal2658. 10.1126/scitranslmed.aal2658 28701474

[48] Oikonomou EK Marwan M Desai MY Mancio J Alashi A Hutt Centeno E Non-invasive detection of coronary inflammation using computed tomography and prediction of residual cardiovascular risk (the CRISP CT study): a post-hoc analysis of prospective outcome data. Lancet. (2018) 392:929–39. 10.1016/S0140-6736(18)31114-0 30170852 PMC6137540

[49] Ueno H Hoshino M Sugiyama T Kanaji Y Nogami K Horie T Pericoronary adipose tissue inflammation on coronary computed tomography in patients with vasospastic angina. JACC Cardiovasc Imaging. (2021) 14:511–2. 10.1016/j.jcmg.2020.08.002 32928704

[50] Yamaguchi M Yonetsu T Hoshino M Sugiyama T Kanaji Y Yasui Y Clinical significance of increased computed tomography attenuation of periaortic adipose tissue in patients with abdominal aortic aneurysms. Circ J. (2021) 85:2172–80. 10.1253/circj.CJ-20-1014 33896902

[51] Rodriguez-Granillo GA Carrascosa P. Detection of coronary inflammation. Lancet. (2019) 393:2198–9. 10.1016/S0140-6736(19)30225-9 31162077

[52] Sui W Li H Yang Y Jing X Xue F Cheng J Bladder drug mirabegron exacerbates atherosclerosis through activation of brown fat-mediated lipolysis. Proc Natl Acad Sci U S A. (2019) 116:10937–42. 10.1073/pnas.1901655116 31085638 PMC6561204

[53] Marso SP Daniels GH Brown-Frandsen K Kristensen P Mann JF Nauck MA Liraglutide and cardiovascular outcomes in type 2 diabetes. N Engl J Med. (2016) 375:311–22. 10.1056/NEJMoa1603827 27295427 PMC4985288

[54] Marso SP Bain SC Consoli A Eliaschewitz FG Jodar E Leiter LA Semaglutide and cardiovascular outcomes in patients with type 2 diabetes. N Engl J Med. (2016) 375:1834–44. 10.1056/NEJMoa1607141 27633186

[55] Gerstein HC Colhoun HM Dagenais GR Diaz R Lakshmanan M Pais P Dulaglutide and cardiovascular outcomes in type 2 diabetes (REWIND): a double-blind, randomised placebo-controlled trial. Lancet. (2019) 394:121–30. 10.1016/S0140-6736(19)31149-3 31189511

[56] Iacobellis G Villasante Fricke AC. Effects of semaglutide versus dulaglutide on epicardial fat thickness in subjects with type 2 diabetes and obesity. J Endocr Soc. (2020) 4:bvz042. 10.1210/jendso/bvz042 32190806 PMC7069837

[57] Morano S Romagnoli E Filardi T Nieddu L Mandosi E Fallarino M Short-term effects of glucagon-like peptide 1 (GLP-1) receptor agonists on fat distribution in patients with type 2 diabetes mellitus: an ultrasonography study. Acta Diabetol. (2015) 52:727–32. 10.1007/s00592-014-0710-z 25577244

[58] Dutour A Abdesselam I Ancel P Kober F Mrad G Darmon P Exenatide decreases liver fat content and epicardial adipose tissue in patients with obesity and type 2 diabetes: a prospective randomized clinical trial using magnetic resonance imaging and spectroscopy. Diabetes Obes Metab. (2016) 18:882–91. 10.1111/dom.12680 27106272

[59] Iacobellis G Camarena V Sant DW Wang G. Human epicardial fat expresses glucagon-like peptide 1 and 2 receptors genes. Horm Metab Res. (2017) 49:625–30. 10.1055/s-0043-109563 28514806 PMC7430146

[60] Beiroa D Imbernon M Gallego R Senra A Herranz D Villarroya F GLP-1 agonism stimulates brown adipose tissue thermogenesis and browning through hypothalamic AMPK. Diabetes. (2014) 63:3346–58. 10.2337/db14-0302 24917578

[61] Dozio E Vianello E Malavazos AE Tacchini L Schmitz G Iacobellis G Epicardial adipose tissue GLP-1 receptor is associated with genes involved in fatty acid oxidation and white-to-brown fat differentiation: a target to modulate cardiovascular risk? Int J Cardiol. (2019) 292:218–24. 10.1016/j.ijcard.2019.04.039 31023563

[62] Zinman B Wanner C Lachin JM Fitchett D Bluhmki E Hantel S Empagliflozin, cardiovascular outcomes, and mortality in type 2 diabetes. N Engl J Med. (2015) 373:2117–28. 10.1056/NEJMoa1504720 26378978

[63] Wiviott SD Raz I Bonaca MP Mosenzon O Kato ET Cahn A Dapagliflozin and cardiovascular outcomes in type 2 diabetes. N Engl J Med. (2019) 380:347–57. 10.1056/NEJMoa1812389 30415602

[64] Requena-Ibanez JA Santos-Gallego CG Rodriguez-Cordero A Vargas-Delgado AP Mancini D Sartori S Mechanistic insights of empagliflozin in nondiabetic patients with HFrEF: from the EMPA-TROPISM study. JACC Heart Fail. (2021) 9:578–89. 10.1016/j.jchf.2021.04.014 34325888

[65] Iacobellis G Gra-Menendez S. Effects of dapagliflozin on epicardial fat thickness in patients with type 2 diabetes and obesity. Obesity. (2020) 28:1068–74. 10.1002/oby.22798 32352644

[66] Sato T Aizawa Y Yuasa S Kishi S Fuse K Fujita S The effect of dapagliflozin treatment on epicardial adipose tissue volume. Cardiovasc Diabetol. (2018) 17:6. 10.1186/s12933-017-0658-8 29301516 PMC5753537

[67] Yagi S Hirata Y Ise T Kusunose K Yamada H Fukuda D Canagliflozin reduces epicardial fat in patients with type 2 diabetes mellitus. Diabetol Metab Syndr. (2017) 9:78. 10.1186/s13098-017-0275-4 29034006 PMC5628447

[68] Fukuda T Bouchi R Terashima M Sasahara Y Asakawa M Takeuchi T Ipragliflozin reduces epicardial fat accumulation in non-obese type 2 diabetic patients with visceral obesity: a pilot study. Diabetes Ther. (2017) 8:851–61. 10.1007/s13300-017-0279-y 28616806 PMC5544615

[69] Bouchi R Terashima M Sasahara Y Asakawa M Fukuda T Takeuchi T Luseogliflozin reduces epicardial fat accumulation in patients with type 2 diabetes: a pilot study. Cardiovasc Diabetol. (2017) 16:32. 10.1186/s12933-017-0516-8 28253918 PMC5335851

[70] Xu L Nagata N Nagashimada M Zhuge F Ni Y Chen G SGLT2 inhibition by empagliflozin promotes fat utilization and browning and attenuates inflammation and insulin resistance by polarizing M2 macrophages in diet-induced obese mice. EBioMedicine. (2017) 20:137–49. 10.1016/j.ebiom.2017.05.028 28579299 PMC5478253

[71] Xu L Nagata N Chen G Nagashimada M Zhuge F Ni Y Empagliflozin reverses obesity and insulin resistance through fat browning and alternative macrophage activation in mice fed a high-fat diet. BMJ Open Diabetes Res Care. (2019) 7:e000783. 10.1136/bmjdrc-2019-000783 31749970 PMC6827766

[72] Xu L Xu C Liu X Li X Li T Yu X Empagliflozin induces white adipocyte browning and modulates mitochondrial dynamics in KK Cg-Ay/J mice and mouse adipocytes. Front Physiol. (2021) 12:745058. 10.3389/fphys.2021.745058 34777009 PMC8578598

[73] Mori Y Terasaki M Hiromura M Saito T Kushima H Koshibu M Luseogliflozin attenuates neointimal hyperplasia after wire injury in high-fat diet-fed mice via inhibition of perivascular adipose tissue remodeling. Cardiovasc Diabetol. (2019) 18:143. 10.1186/s12933-019-0947-5 31672147 PMC6823953

[74] Rajeev SP Cuthbertson DJ Wilding JP. Energy balance and metabolic changes with sodium-glucose co-transporter 2 inhibition. Diabetes Obes Metab. (2016) 18:125–34. 10.1111/dom.12578 26403227

[75] Wilcox T De Block C Schwartzbard AZ Newman JD. Diabetic agents, from metformin to SGLT2 inhibitors and GLP1 receptor agonists: JACC focus seminar. J Am Coll Cardiol. (2020) 75:1956–74. 10.1016/j.jacc.2020.02.056 32327107 PMC7219531

[76] Pi-Sunyer X Astrup A Fujioka K Greenway F Halpern A Krempf M A randomized, controlled trial of 3.0 mg of liraglutide in weight management. N Engl J Med. (2015) 373:11–22. 10.1056/NEJMoa1411892 26132939

